# Availability and Quality of Physical Activity Resources in Neighborhood Parks for Pregnant Women and Women of Childbearing Age

**DOI:** 10.5888/pcd15.170411

**Published:** 2018-06-14

**Authors:** Sophia S. Leggett, Joan M. Wesley, Anitha Myla, Pamela McCoy, Nicole Betson

**Affiliations:** 1 Department of Behavioral and Environmental Health, School of Public Health, Center of Excellence in Minority Health and Health Disparities, Jackson State University, Jackson, Mississippi; 2 Department of Urban and Regional Planning, College of Public Service, Jackson State University, Jackson, Mississippi; 3 Department of Epidemiology and Biostatics, School of Public Health, Jackson State University, Jackson, Mississippi; 4 Jackson Heart Study Community Outreach Center, School of Public Health, Jackson State University, Jackson, Mississippi

## Abstract

Neighborhood parks help women engage in physical activity (PA). We used the physical activity resources assessment instrument to determine the availability, quality and quantity of physical features, and amenities in 19 neighborhood parks randomly selected from the Jackson, Mississippi, Metropolitan Statistical Area. Madison County averaged the most quality PA features (mean, 13) and quality PA amenities (mean, 25.8), and it averaged the least quality incivilities (mean, 1.6). The total neighborhood parks quality physical activity resources (QPAR) was determined by a composite index QPAR of features, amenities, and incivilities. Neighborhood parks’ QPAR index was 545 (mean, 28.7), showing less use of parks. Quality PA features were significantly (*P* < .01) associated with quality PA amenities.

## Objective

In 2013, the overall adult obesity rate in Mississippi was 35.1%, and the rate for women was 36.4%. The obesity prevalence was higher for black residents (42.5%) compared with white residents (31.1%) (1). Hinds County is mostly black (72%), Rankin County is mostly white (77%), and Madison County is more than half white (57%) (2). Obesity prevention among women of childbearing age (15–44 years) is strongly associated with regular physical activity (3). Increased physical activity among women during pregnancy could reduce risk of maternal obesity, gestational diabetes, cesarean delivery, and preeclampsia (4,5). The objectives of this study were to examine the availability of physical activity resources (PARs) and assess the quality physical activity resources (QPAR) index of the selected neighborhood parks for pregnant women and women of childbearing age to improve their overall health.

## Methods

The Pregnancy and Early Life in the South (PEARLS) project was used with the questionnaire adapted from the validated Physical Activity Resources Assessment Instrument (PARA) (6). A Google search of public state and local city parks, and recreation amenities was generated. To identify the parks, women aged 15 to 44 signed for each precoded PARA questionnaire to assess each park. Nineteen parks in the Jackson Metropolitan Statistical Area (Hinds, Madison, and Rankin counties) were randomly selected by zip code from 64 parks. The locations of parks were confirmed using ground truthing (ie, people on the ground accurately confirming the location of each park). Parks were assigned identification numbers based on the city and county in which they were located. A trained research team collected data from May through August 2014.

PARA was used to determine parks’ availability, quality and quantity of physical features, and amenities. Researchers counted and rated 25 park elements, including 13 physical activity features and 12 amenities. Features and amenities were rated for quality from 1 to 3 (1 = poor; 2 = fair; 3 = good). Incivilities, such as overgrown grass, included 13 elements that also were coded from 1 to 3 (1 = little; 2 = medium; 3 = a lot) (6). Environmental scans were conducted during daylight hours and completed in 30 to 45 minutes. PA features in each park were assessed for availability and quality. Amenities were assessed for the availability and quality of each park.

We used descriptive analyses to evaluate the quality of features and amenities, and we determined a composite index individual QPAR. For each park, the quality score was based on the sum of the quality features and amenities ratings, minus the incivilities ratings (7). To aggregate the score at the county level, the quality score for each park was summed then divided by the total number of parks. We used the Pearson correlation coefficient to assess the association between the PA quality features, amenities, incivilities, and QPAR index. Significance was set at *P* <.05. All analyses were conducted using SPSS 22.0 (IBM Corporation).

## Results

On average, Madison neighborhood parks had more quality PA features (mean, 13) than the parks in Hinds County (mean, 9.4) and Rankin County (mean, 9.2) (Table 1). Similarly, Madison neighborhood parks had more quality amenities (mean, 25.8) than those in Rankin County (mean, 19), and Hinds County (mean, 18.4). Parks of better quality had higher features and amenities scores and lower incivility scores.

**Table 1:** Mean Quality Ratings and QPAR Index for Physical Activity Features and Amenities of Parks (N = 19), Jackson, Mississippi, 2014

County/Park Identification	Quality of Features	Quality of Amenities	Quality of Incivilities	QPAR Index
**Madison**				
RM1	15	28	2	41
RM2	6	25	2	29
RM3	12	26	1	37
MM4	21	27	3	45
MM5	11	23	0	34
Total	65	129	8	186
Mean (SD)	13 (5.5)	25.8 (1.9)	1.6 (1.1)	37.2 (6.2)
**Rankin**				
RP6	11	21	1	31
RP7	21	26	0	47
RP8	15	27	3	39
RB9	9	15	3	21
RB10	3	9	0	12
RB11	3	8	0	11
RB12	3	27	7	23
Total	65	133	14	184
Mean (SD)	9.2 (7.0)	19 (8.4)	2 (2.6)	26.3 (13.5)
**Hinds**				
HJ13	0	10	7	3
HJ14	10	13	1	22
HJ15	7	15	3	19
HJ16	9	18	3	24
HC17	14	21	3	32
HC18	15	31	2	44
HC19	11	21	1	31
Total	66	129	20	175
Mean (SD)	9.4 (5.0)	18.4 (6.9)	2.8 (2.0)	25 (12.7)
Total	196	391	42	545
Mean (SD)	10.3 (6.3)	20.5 (8.5)	2.3 (2.1)	28.7 (12.3)

Abbreviations: HC, Hinds-Clinton; HJ, Hinds-Jackson; MM, Madison-Madison; QPAR, quality physical activity resources; RB, Rankin-Brandon; RM, Ridgeland-Madison; RP, Rankin-Pearl; SD, standard deviation.

Of the neighborhood parks, 79% (n = 15) had incivilities. Hinds County had the largest number of incivilities (mean, 2.8), followed by Rankin County (mean, 2.0) and Madison County (mean, 1.6). Overgrown grass was the most frequently reported incivility (42%, n = 8) for the parks. Litter and auditory annoyances were the second most frequently noted incivilities (31%, n = 6). There was no evidence of vandalism, sex paraphernalia, graffiti, or unattended dogs in any of the neighborhood parks.

QPAR index scores ranged from 3 to 47 for all 19 parks, with a total QPAR index of 545 (mean, 28.7). Madison County had the highest QPAR index (mean, 37.2) followed by Rankin County (mean, 26.3), and Hinds County (mean, 25.0) (Table 1). Quality features and quality amenities were significantly associated with QPAR score (*P* < .01). Quality PA features and quality amenities were also significantly associated with QPAR score (*P* < .01) (Table 2).

**Table 2:** Correlations Between Physical Activity Characteristics and Physical Activity Resource Quality,^a^ Jackson, Mississippi, 2014

	QPAR Index	Number of PARs	Features Score	Amenities Score	Incivilities Score
**QPAR Index**	**1**				
Number of PARs	−0.334	1			
Features score	0.857^b^	−0.18	1		
Amenities score	0.830^b^	−0.288	0.554^c^	1	
Incivilities score	−0.128	−0.006	−0.267	0.212	1

Abbreviations: PAR, physical activity resource; QPAR, quality of physical activity resources score.

a QPAR index consists of resource features, amenities, and incivilities scores; and the PAR (8).

b *P* < .01.

c *P* < .05.

## Discussion

Neighborhood public parks may help facilitate PA. They provide places for people to walk or jog, and many have facilities for sports, exercise, and vigorous activity (9). Physical activity plays a major role in the overall health of women of childbearing age. Physical exercise during pregnancy improves fitness and pregnancy-related outcomes (10). Select neighborhood parks in the Jackson, Mississippi, MSA were assessed for PAR availability and quality. Greater access to high-quality parks may substantially increase rates of physical activity among pregnant women and women of childbearing age.

Parks in the study had good quality features and amenities; however, incivilities such as overgrown grass were consistently present. The quality of PARs, with greater PA features and amenities combined with fewer incivilities, may be an important consideration for engaging in physical activity (8,11). Madison County parks had the highest QPAR (mean, 37.2) of the 3 studied counties. The parks with higher QPAR scores indicate greater physical activity.

Although the findings are generalizable to women of childbearing age, the quality of the data and consistency of the findings inform research and practice among other populations. According to previous studies, intervening during adolescence to promote healthy weight throughout a woman’s reproductive years may improve maternal preconception health (12).

Limitations of this study include small sample size of neighborhood parks by zip code and the variation in the number of parks by county. The study’s strengths included randomized selections of neighborhood parks and the use of a reliable and validated PARA instrument by trained researchers.

Future research should identify how to motivate women to be physically active and how to improve their overall health. Health maintenance is needed to improve maternal health and reduce the risk for maternal obesity, gestational diabetes mellitus, and preeclampsia. Research in this area could assist policy makers and political leaders in work with communities to update the quality of publicly available PARs. The study’s implication for public health practice is to help health care professionals encourage women and pregnant women to engage in PA for improving their overall health.
